# GC/MS Analysis, Cytotoxicity, and Antiviral Activities of *Annona glabra* Hexane Extract Supported by In Silico Study

**DOI:** 10.3390/molecules28041628

**Published:** 2023-02-08

**Authors:** Dalia M. Soleman, Omayma A. Eldahshan, Mona H. Ibrahim, Hanan A. Ogaly, Heba M. Galal, Gaber El-Saber Batiha, Rawah H. Elkousy

**Affiliations:** 1 Department of Pharmacognosy, Faculty of Pharmacy, Misr International University, Cairo P.O. Box 41611, Egypt; 2 Department of Pharmacognosy, Faculty of Pharmacy, Ain Shams University, Cairo 11566, Egypt; 3 Center for Drug Discovery Research and Development, Faculty of Pharmacy, Ain Shams University, Cairo 11566, Egypt; 4 Department of Pharmaceutical Medicinal Chemistry and Drug Design, Faculty of Pharmacy (for Girls), Al-Azhar University, Nasr City, Cairo 11651, Egypt; 5 Chemistry Department, College of Science, King Khalid University, Abha 61421, Saudi Arabia; 6 Biochemistry and Molecular Biology Department, Faculty of Veterinary Medicine, Cairo University, Giza 12211, Egypt; 7 Department of Medical Physiology, College of Medicine, Jouf University, Sakaka 72388, Saudi Arabia; 8 Department of Medical Physiology, Faculty of Medicine, Assiut University, Assiut 71515, Egypt; 9 Department of Pharmacology and Therapeutics, Faculty of Veterinary Medicine, Damanhour University, Damanhour 22511, Egypt; 10 Department of Pharmacognosy, Faculty of Pharmacy (for Girls), Al-Azhar University, Nasr City, Cairo 11651, Egypt

**Keywords:** *Annona glabra*, cytotoxicity, antiviral activity, GC/MS, docking screening

## Abstract

*Annona glabra* Linn is employed in conventional medicine to treat a number of human disorders, including cancer and viruses. In the present investigation, the significant phytochemical components of *Annona glabra* hexane extract were identified using gas chromatography–mass spectrometry (GC-MS) analysis. Three major compounds were identified in the hexane extract: tritriacontane (30.23%), 13, 17-dimethyl-tritriacontane (22.44%), and limonene (18.97%). MTT assay was used to assess the cytotoxicity of the extract on six human cancer cell lines including liver (HepG-2), pancreas (PANC-1), lung (A-549), breast (MCF-7, HTB-22), prostate (PC-3), and colon (CACO-2, ATB-37). The extract exhibited significant cytotoxic activity against both CACO-2 and A-549 cancer cell lines (IC_50_ = 47 ± 0.74 μg/mL and 56.82 ± 0.92 μg/mL) in comparison with doxorubicin (IC_50_ = 31.91 ± 0.81 μg/mL and 23.39 ± 0.43 μg/mL) and of SI of 3.8 and 3.1, respectively. It also induced moderate-to-weak activities against the other cancerous cell lines: PC-3, PANC-1, MCF-7, and HepG-2 (IC_50_ = 81.86 ± 3.26, 57.34 ± 0.77, 80.31 ± 4.13, and 57.01 ± 0.85 μg/mL) in comparison to doxorubicin (IC_50_ = 32.9 ± 1.74, 19.07 ± 0.2, 15.48 ± 0.84 and 5.4 ± 0.22 μg/mL, respectively) and SI of 2.2, 3.1, 2.2, and 3.1, respectively. In vitro anti-HSV1 (Herpes simplex 1 virus) and HAV (Hepatitis A virus) activity was evaluated using MTT colorimetric assay with three different protocols to test protective, anti-replicative, and anti-infective antiviral activities, and three separate replications of each experiment were conducted. The plant extract showed promising protective and virucidal activity against HSV1 with no significant difference with acyclovir (79.55 ± 1.67 vs. 68.44 ± 7.62 and 70.91 ± 7.02 vs. 83.76 ± 5.67), while it showed mild protective antiviral activity against HAV (48.08 ±3.46) with no significant difference vs. acyclovir (36.89 ± 6.61). The selected main compounds were examined for their bioactivity through in silico molecular docking, which exhibited that limonene could possess the strongest antiviral properties. These findings support *Annona glabra’s* conventional use, which is an effective source of antiviral and anticancer substances that could be used in pharmaceuticals.

## 1. Introduction

For centuries, cultures around the world have learned how to use herbal medicine to improve healthcare regimens. The importance of plant-based products for disease treatment is growing exponentially due to the increased incidence of adverse drug reactions and the development of microbial resistance to available antimicrobial drugs [1]. Natural products possess tremendous potential for medical development. Compounds originated from plants and their semisynthetic, synthetic, and analogous components have helped mankind as a primary source of medications for treating human illnesses. Therefore, for treating disease states where drug therapy is a reasonable course of action, plant materials are acceptable beginning points for developing novel agents [2]. Herbal medicines have a wide range of activities on the human body, including hepatoprotective [3,4], antidiabetic, anti-inflammatory [5], antioxidant, chemoprevention, immunoregulatory, and anti-toxic activities [6]. However, users should consider efficacy, quality, and safety when using natural remedies. A paradigm based on ethnobotanical and ethnopharmacological data would be more advantageous and cost-effective in the viral and cancer drug discovery program for discovering potential antiviral and anticancer molecules than the mass screening of plant species [2].

The family Annonaceae includes the genus *Annona*, which is a genus of tropical fruit trees. Annonaceae is a family of flowering plants that includes trees and shrubs. Several genera yield fruit that can be consumed, most notably *Annona* [7]. The family’s most significant genus is this one, *Annona*, which includes approximately 119 species [8]. After *Guatteria*, it is regarded as the second-largest genus of flowering plants in the family Annonaceae [9]. The Latin word “anon,” which means “yearly produce” and refers to the production of fruits by many species in this genus, is the source of the name “*Annona*” [10]. *Annona glabra* Linn, commonly named as pond apple [2], is a Florida-native tropical fruit tree (United States of America). It is well recognized that the *Annona* plant has medicinal properties, including antidiarrheal [11], antiparasitic [12], anticonvulsant [13], antiulcer [14], antioxidant [15], antidiabetic [16], antidepressant [17], antiviral [18], Dengue vector control [19], anticancer [20], and anti-inflammatory [21] activities. In addition, pond apple leaves and bark have historically been employed as insect and parasiticides [12]. The complete plant of the pond apple tree is used as an anticancer medication in China, and the leaves are used to cure chronic bronchitis [2]. An alcoholic seed extract from *Annona glabra* has demonstrated anticancer action, making it a viable source of chemicals for cancer treatment [22]. From the phytochemical standpoint, the Annonaceous acetogenins, a novel class of chemicals discovered from *Annona* plants and showing potent anticancer activity, were found to be abundant in the plant. Three different *Annona* plant extracts—bullatacin, asinicin, and bullatacinone—showed 300-, 80-, and 40-times more anticancer effects than taxol in vivo tests on mice with leukemia. Liu et al. proved the anticancer effect of two extracts from *Annona glabra*, which is 100,000 times higher than adriamycin. *Annona* plants have emerged as a new, promising source for cutting-edge cancer treatments [20]. *A. glabra*’s phytochemical study isolated acetogenins, ent-kauranes, and alkaloids [23]. Cunabic acid and ent-Kauran-19-al-17-oic acid, two diterpenoid substances derived from *Annona glabra* Linn, can clearly stop the growth of HLC cell line SMMC-7721 [20].

Glacin A and glacin B, two novel bioactive mono-THF acetogenins, have shown strong and targeted in vitro cytotoxicities against a variety of human solid cancer cell lines [24]. The hexane extract of *A. glabra* stem bark also revealed significant antibacterial activity in a preliminary screening [22]. The fruits of *Annona glabra* showed some action against HIV replication in H9 lymphocyte cells and considerable suppression of HIV-reverse transcriptase through phytochemical analyses [25].

The increase in virus infections and cancer diseases has necessitated the ever-increasing demand for therapeutic natural product drugs due to the lack of vaccines, the emergence of viral strains resistant to antiviral medications, and the side effects of cancer chemotherapy. Antiviral activity is not widely reported from *Annona* species. In light of the fact that acyclovir, a nucleoside analog, is one of the medications that is endorsed for the treatment of HSV infections, our curiosity to assess the antiviral activity of *Annona glabra* against some notable and easily cultivable viral pathogens, such as Hepatitis A virus (HAV), Herpes simplex type 1 (HSV1), and New Castle with three different mechanisms, has been peaked.

Herein, using gas chromatography–mass spectrometry (GC-MS), we extracted and specified some of the components of the hexane extract of *Annona glabra* leaves and assessed the cytotoxic activity of *A. glabra* against hepatocellular carcinoma (HepG-2), colorectal adenocarcinoma (CACO-2), breast cancer (MCF-7), lung (A-549), prostate (PC-3), and pancreas cancer (PANC-1) cell lines. One chemotherapy drug used to treat cancer is called doxorubicin was used as a positive control. Through intercalation and suppression of macromolecular production, it interacts with DNA. This prevents topoisomerase II, an enzyme that loosens DNA supercoils for transcription, from progressing. Additionally, it keeps the DNA double helix from being released and stops the replication process by stabilizing the topoisomerase II complex after it has broken the DNA chain for replication [26].

## 2. Results

### 2.1. GC/MS Analysis Identification and Quantification of the Chemical Constituents

**Figure 1:**
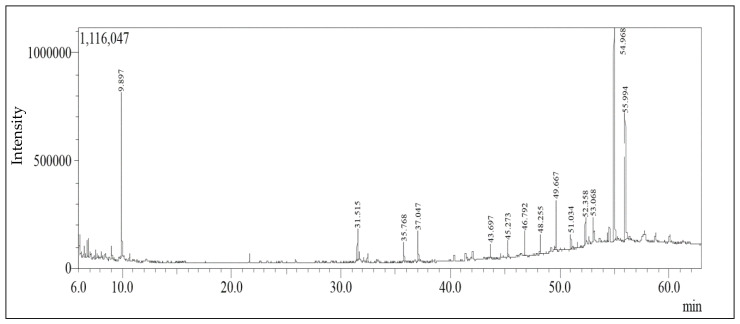
GC/MS chromatogram (TIC) of *Annona glabra* hexane extract.

The chromatogram of GC-MS spectra analysis (Figure 1, Table 1) exhibits 14 peaks of the hexane extract compounds of *Annona glabra* leaves. The main components were tritriacontane (30.23%), 13,17-dimethyl- tritriacontane (22.44%), and limonene (18.97%). The identification was made using the mass spectral data (molecular ion peaks, fragmentation patterns), Kovats retention index (RI), as well as published data from the Pherobase website, Adams (version 2007), NIST Mass Spectral Library (December 2005) [27,28], and other sources.

### 2.2. In Vitro Cytotoxicity Assay

The MTT test was used to assess the cytotoxicity of *Annona glabra* hexane extract against normal Vero and six human cancer cell lines of the liver (HepG-2), breast (MCF-7), colon (CACO-2), pancreas (PANC-1), lung (A-549) and prostate (PC-3). The cytotoxicity experiment was performed 48 h after the extract was inoculated with various doses ranging from 31.25 to 1000 µg/mL. The outcomes revealed that the sample had noncytotoxic action to the normal cells (IC_50_ value > 100 µg/mL). Moreover, the investigated sample, in a way depending on concentration, prevented the proliferation of the tumor cells. Due to this, SI of the extract to Vero and different cancer cells was more than that of doxorubicin >1, indicating that the extract was safer. The safer the medicine, the higher the SI value.

The IC_50_, μg/mL (the concentration that results in a 50% reduction in cell viability), and selectivity index (SI) values were used to express the sensitivity of the cell lines to the sample. In general, the drug potency is inversely proportional to the IC_50_ values.

The easiest evaluation of IC_50_ is to plot x-y and fit the data with a straight line (linear regression). IC_50_ value is then evaluated using the fitted line, i.e.,

Y = a ∗ X + b,

IC_50_ = (0.5 − b)/a.

#### Cytotoxic Effect of *Annona* Extract

The cytotoxic activity of *Annona* extract in comparison with doxorubicin against Vero cell line is 179.88 ± 0.28 μg/mL and 34.26 ± 0.55 μg/mL, respectively.

Table 2 showed that the extract exhibited significant cytotoxic activity against both CACO-2 and A-549 cancer cell lines (IC_50_ = 47 ± 0.74 μg/mL and 56.82 ± 0.92 μg/mL) in comparison with doxorubicin (IC_50_ = 31.91 ± 0.81 μg/mL and 23.39± 0.43 μg/mL) and of SI of 3.8 and 3.1, respectively. It also induced moderate-to-weak activities against the other cancerous cell lines, i.e., PC-3, PANC-1, MCF-7, and HepG-2 (IC_50_ = 81.86 ± 3.26, 57.34 ± 0.77, 80.31 ± 4.13, and 57.01 ± 0.85 μg/mL) in comparison to doxorubicin (IC_50_ = 32.9± 1.74, 19.07 ± 0.2, 15.48± 0.84 and 5.4 ± 0.22 μg/mL, respectively) and SI of 2.2, 3.1, 2.2, and 3.1, respectively.

### 2.3. Antiviral Assay

#### 2.3.1. *Annona glabra* Sample MNTC Determination

The MNTC is known as the maximum nontoxic concentration required to produce optimum biological activity and less of a cytotoxic effect. As shown in Table 3, MNTC was determined for *Annona* hexane extract as 7.81 μg/mL vs. acyclovir (62.5 μg/mL) as a reference drug.

#### 2.3.2. Antiviral Efficacy of the *Annona glabra* Extract versus HAV and HSV1

The Minimum nontoxic concentration of *Annona glabra* sample was tested against each virus to assess the mechanism behind its antiviral action on the basis of three protocols (Table 4, Table 5 and Table 6).

##### Comparison of the Effect of the Extract versus HSV1 vs. Acyclovir with Different Protocols

The antiviral action of acyclovir was demonstrated in the following order: protocol B (92.69 ± 1.32) > protocol A (83.76 ± 5.67) > protocol C (68.44 ± 7.62), as shown in Table 5. On investigating the antiviral performance of *Annona glabra* extract versus HSV1, it was discovered that the extract showed pronounced antiviral activity with HSV1 using protocol C and protocol A, with no significant difference with acyclovir (79.55 ± 1.67 vs. 68.44 ± 7.62) and (70.91 ± 7.02 vs. 83.76 ± 5.67), and moderate activity with protocol B (61.91 ± 3.51), with no significant difference. Acyclovir showed the highest activity with protocol B (92.69 ± 1.32). Thus, the *Annona* extract showed pronounced protective and virucidal antiviral activity against HSV1, and moderate anti-replicative activity with no significant difference.

##### Comparison of the Effect of the *Annona* Extract versus HAV vs. Acyclovir with Different Protocols

The antiviral action of acyclovir was demonstrated in the following order: protocol B (54.8± 11.7) > protocol A (46.17 ± 1.67) > protocol C (36.89 ± 6.61), as shown in Table 6. On investigating the antiviral performance of *Annona* extract versus HAV (Table 6), it was found that, for dealing with the hexane extract, HAV’s antiviral activity was: protocol C (48.08± 3.46) > protocol A (36.81 ± 2.67), with no significant difference. Furthermore, the least among the protocols was protocol B (20.13 ± 3.1), which showed no antiviral activity against HAV. Thus, *Annona* extract showed mild protective antiviral activity against HAV.

### 2.4. Molecular Docking

#### 2.4.1. Docking into Cyclin-Dependent Kinase 2 Active Site

**Figure 2:**
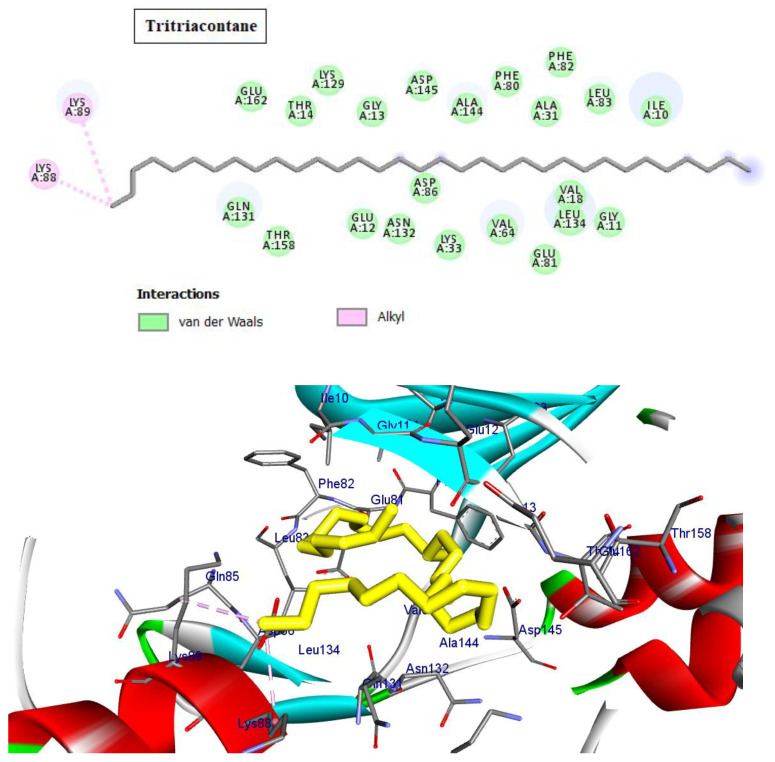
Two-dimensional and three-dimensional images of tritriacontane docked into the ATP binding site CDK-2 enzyme.

**Figure 3:**
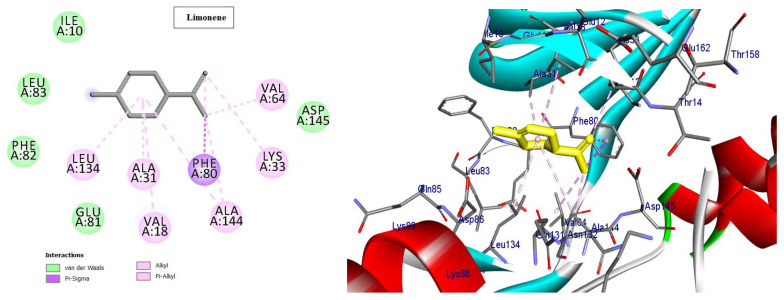
Two-dimensional and three-dimensional images of limonene docked into the ATP binding site CDK-2 enzyme.

**Figure 4:**
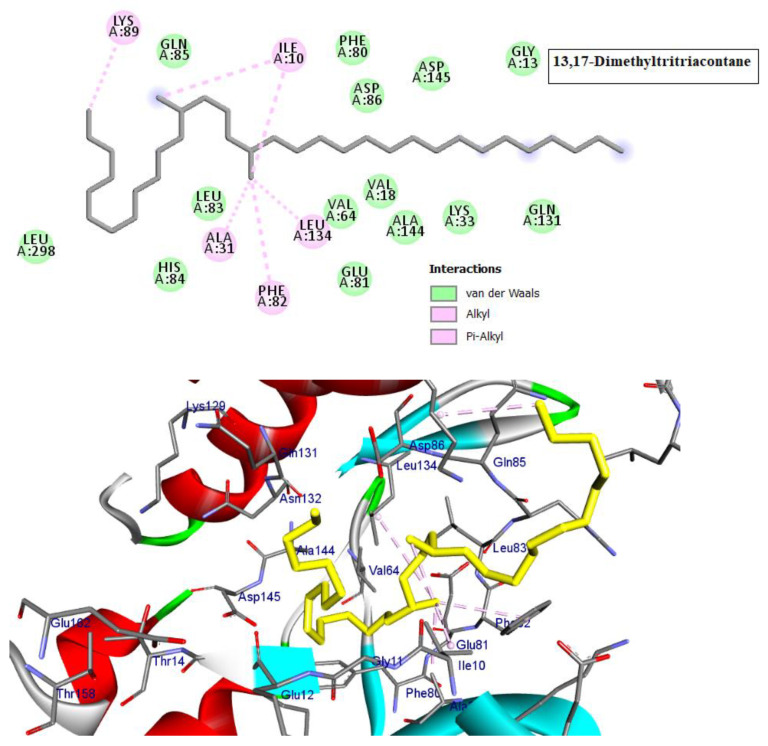
Two-dimensional and three-dimensional images of 13,17-dimethyltritriacontane docked into the ATP binding site CDK-2 enzyme.

**Figure 5:**
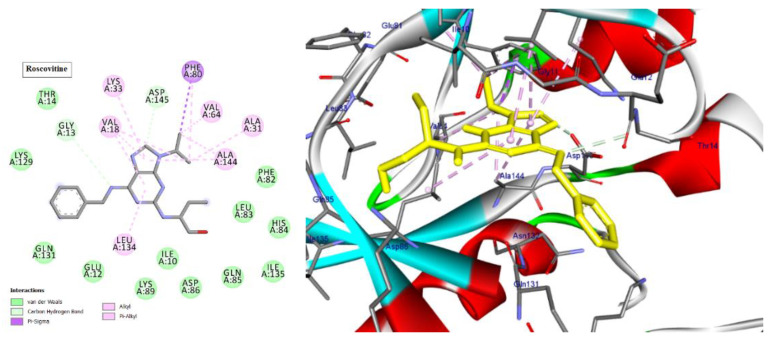
Two-dimensional and three-dimensional images of cocrystal ligand (Roscovitine) docked into the ATP binding site CDK-2 enzyme.

**Figure 6:**
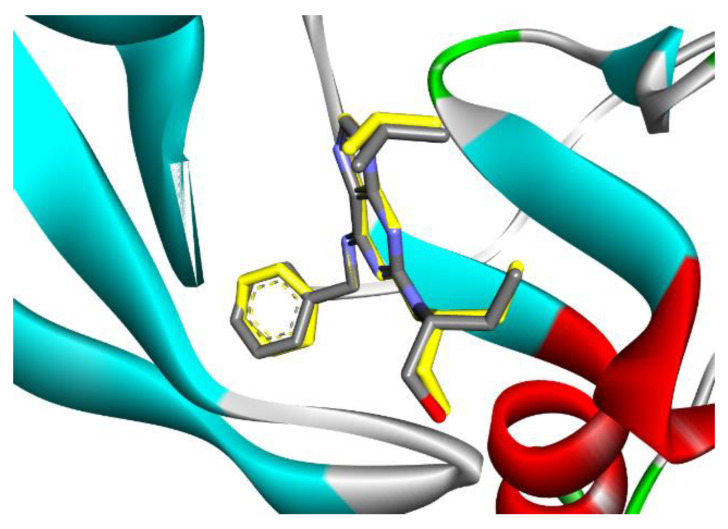
The superposition of cocrystal ligand (Roscovitine) with its docked pose showed an appreciable RMSD of 0.48 Å.

**Figure 7:**
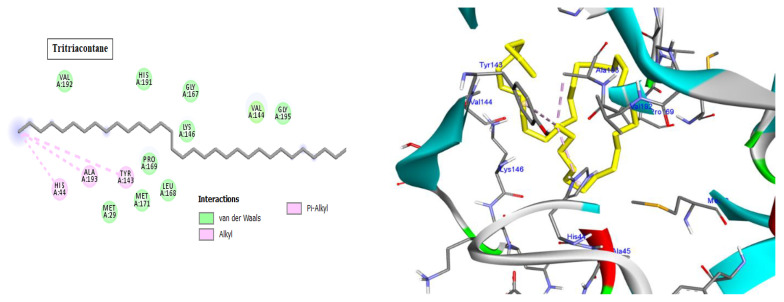
Two-dimensional and three-dimensional images of tritriacontane docked into the HAV 3C protease enzyme binding site.

**Figure 8:**
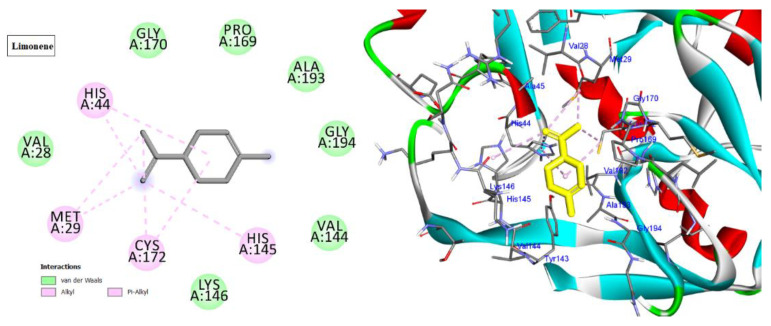
Two-dimensional and three-dimensional images of limonene docked into the HAV 3C protease enzyme binding site.

Cyclin-dependent kinases (CDKs) are a class of serine/threonine kinases that work with cyclins to ensure the development of the cell cycle [29]. CDKs are the key managers of cell division and are capable of creating a heterodimer with cyclins to catalyze the phosphorylation of diverse substrates, resulting in the change of the cell cycle at different phases [30]. Intriguingly, CDKs have significant roles in antiviral responses; the present investigation targeted CDK2 as a potential therapeutic target in an effort to develop drugs with antiviral efficacy against HSV-1. Tritriacontane, limonene, and 13,17-dimethyltritriacontane, the major compounds of hexane extract, were docked and linked to the ATP binding site of the CDK2 enzyme as roscovitine, which is a cocrystal ligand (Figure 2, Figure 3, Figure 4, Figure 5, Figure 6, Figure 7 and Figure 8; Table 7). Tritriacontane forms two alkyl bonds with the essential binding residues Lys89 and Lys88 [31] (Figure 2; docking scores −6.6 kcal/mol). 13,17-dimethyltritritritriacontane exhibited a favorable interaction by establishing six hydrophobic bonds in the CDK2 active site (Figure 4). The binding mode of limonene in the active site of CDK2 kinase is depicted in Figure 8, with the highest docking score of −7.5 kcal/mol coming from roscovitine. Limonene docked similarly to roscovitine (Figure 5) at the hinge region of CDK2 and interacts hydrophobically with key residues, including Val18, Lys33, Ala31, Val64, Leu134, and Ala144 [31]. Additionally, it interacts with the gatekeeper residue Phe80 through a Pi-sigma interaction. Figure 6 displayed a significant RMSD of 0.48 Å for the cocrystal ligand (roscovitine) in its docked position.

#### 2.4.2. Docking into Hepatitis A Virus 3C (HAV 3C) Protease Enzyme Active Site

**Figure 9:**
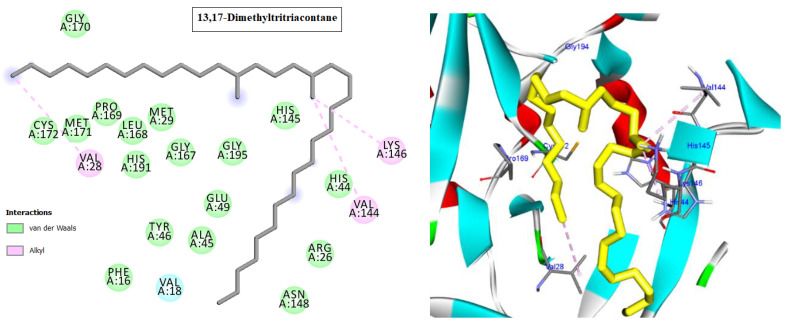
Two-dimensional and three-dimensional images of 13,17-dimethyltritriacontane docked into the HAV 3C protease enzyme binding site.

**Figure 10:**
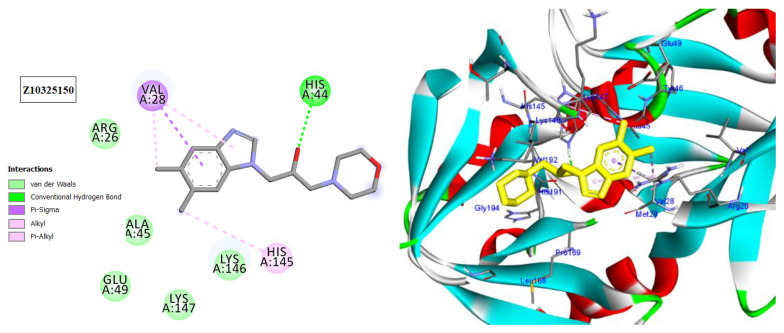
Two-dimensional and three-dimensional images of inhibitor drug (Z10325150) docked into the HAV 3C protease enzyme binding site.

The HAV 3C protease (3Cpro) is in control of the majority of cleavages within the viral polyprotein, allowing for viral replication and propagation. HAV 3Cpro is one of the primary targets for the development of anti-HAV medications due to its critical role in viral replication and proliferation [32]. The three docked compounds and the inhibitor drug (Z10325150) were anchored in the catalytic site, with docking scores ranging from −6.3 to −6.9 kcal/mol (Figure 7, Figure 8, Figure 9 and Figure 10; Table 7). All of them interact with His44, except for 13,17-dimethyltritriacontane (Figure 7, Figure 8, Figure 9 and Figure 10), which displayed three hydrophobic interactions with Lys146, Val144, and Val28 (Figure 9). Limonene formed seven alkyl bonds with His44, Cys172, His145, and Met29 (Figure 8), which showed the highest docking score of −6.9 kcal/mol coming from the Z10325150 compound.

## 3. Discussion

Viral infections continue to be a major cause of morbidity and mortality around the world. With more organ transplants, blood transfusions, and the use of hypodermic syringes, the number of patients diagnosed with viral infections rises alarmingly each year. Traditional antiviral drugs, such as ribavirin and interferon, are effective in vitro against most viruses but are frequently ineffective in patients. There is currently no approved treatment for many types of viruses, and vaccination is limited to mumps, hepatitis A, and varicella [33]. Furthermore, in most cases, their use is accompanied by the appearance of side effects or the formation of resistant viral mutants, making therapy ineffective. Therefore, turning to nature to find effective therapies is a good solution to this problem. Thus, the need to discover novel antiviral herbal remedies has been mandated [1]. Numerous studies have been conducted on the cytotoxic and antiviral activities of natural products. In this study, the GC-MS investigation of *Annona* extract showed the presence of 14 phytochemical compounds, in which tritriacontane, limonene, and 13,17-dimethyltritriacontane are the major peaks.

The investigated extract exhibited a broad cytotoxic activity range against the cancer cell lines under examination. Such differences in cytotoxicity between different types of cells can be explained owing to the variations in their morphology, genomes, and origins, which lead to variations in how susceptible they are to chemotherapy. Thus, the pronounced cytotoxicity of the examined extract is a direct result of the high concentration of compounds in the plant extract. The extract exhibited significant cytotoxic activity against both CACO-2 and A-549 cancer cell lines in comparison with doxorubicin and SI of 3.8 and 3.1, respectively. It also induced moderate-to-weak activities against the other cancerous cell lines, i.e., PC-3, PANC-1, MCF-7, and HepG-2, when compared to doxorubicin and SI of 2.2, 3.1, 2.2, and 3.1, respectively. High selectivity was achieved when the SI was ≥ 3. As the selective index (SI) demonstrates the differential activity of the sample, the greater the SI value is, the more selective it is. An SI value less than two indicates general toxicity of the sample [22]. So, the extract exhibited no cytotoxic activity against normal cells and is considered selective to CACO-2, A-549, PANC-1, and HepG-2.

Annoglabayin, an innovative *Annona* dimeric kaurane diterpenoid, causes apoptosis in HepG-2 cells by altering the mitochondria [34]. Annoglacins A and B were 1000- and 10,000-times more powerful than adriamycin against human pancreatic cancer (PANC-2) and breast carcinoma (MCF-7) cell lines, respectively [35]. Annoglabasin H exhibited significant cytotoxic activity on the MCF-7, SK-Mel2, LU-1, MCF-7, and KB human cancer cell lines [36]. *A. glabra* had a substantial amount of cytotoxic activity against the HL-60 cell line, with an IC_50_ value of 9.0 ± 1.0 µM, but not against the normal Hel-299 cell line [23]. Cunabic acid and ent-kauran-19-al-17-oic acid, two diterpenoid chemicals isolated from *Annona glabra* Linn, can clearly stop the growth of the HLC cell line (SMMC-7721) [20].

Moreover, we assessed the plant extract’s antiviral capacity and mechanism of action, which was designed to measure the protective, anti-replicative, and anti-infective activity. The MNTC of plant extract vs. acyclovir, a reference medicine utilized as a positive control, was employed in all antiviral assays.

This study proved that the plant extract showed promising protective and virucidal activity against HSV1 (79.55 ± 1.67 and 70.91 ± 7.02) with no significant differences with acyclovir, while the plant extract showed moderate anti-replicative activity against HSV1 (61.91 ± 3.51) with no significant difference. Moreover, the plant extract showed mild protective antiviral activity against HAV (48.08 ± 3.46) with no significant difference vs. acyclovir (36.89 ± 6.61) and no anti-replicative antiviral activity versus HAV (20.13 ± 3.1).

The results offer experimental proof that *Annona glabra* extract exhibited higher antiviral efficacy versus HSV1 than HAV for protocol C and A. Acyclovir, as a positive control, is one of the medications that has been approved for use as a first-line treatment for HSV infections. It works by blocking DNA synthesis and viral DNA polymerase by targeting viral thymidine kinase, which prevents viral replication [37]. These activities are correlated to the compounds with high concentrations inside the plant extract. From previous studies, it was reported that *Annona glabra* displayed mild activity versus HIV replication in H9 lymphocyte cells and considerable inhibition of HIV-reverse transcriptase [25]. Moreover, Maqian essential oils showed promising antiviral activities owing to the presence of L-limonene and D-limonene [38]. Essential Oils from Citrus *Aurantium* L. have virucidal activity against Influenza A Virus H1N1 due to the presence of D- and L-limonene, which may be employed as an efficient disinfectant against viruses [39]. Monoterpenes beta-pinene and limonene have been reported to show antiherpetic activity and might be used as powerful antiviral agents in recurrent herpes infections [40].

In silico molecular docking is one of the best techniques to determine new ligands for proteins with known structure and thus plays a significant role in the development of drugs based on structure [41]. Intriguingly, CDKs have significant roles in antiviral responses; a recent publication review summarizes the actions of CDKs in antiviral innate immunity. CDKs are potential cellular targets, as they are implicated in the transcription and replication of viral genomes [42,43]. Since CDK inhibitors have manageable side effects in clinical studies for the treatment of cancer and other disorders, they have been investigated as antivirals. Antigen expression study indicates that latent HSV1 reactivation is associated with CDK2 expression in neurons. It has shown that roscovitine (CDK2 inhibitor) inhibits the reactivation of HSV-1 in ex vivo neurons. On the basis of this information, the present investigation targeted CDK2 as a potential therapeutic target in an effort to develop drugs with antiviral efficacy against HSV-1 [44]. Tritriacontane, limonene, and 13,17-dimethyltritriacontane were docked and linked to the ATP binding site of the CDK2 enzyme as roscovitine, which is a cocrystal ligand. Tritriacontane forms two alkyl bonds with docking scores −6.6 kcal/mol [31]. The binding mode of limonene in the active site of CDK2 kinase had the highest docking score −7.5 kcal/mol, which came from roscovitine. Limonene was docked similarly to roscovitine at the hinge region of CDK2 and interacts hydrophobically with key residues. 13,17-dimethyltritriacontane and tritriacontane exhibited less binding activity than limonene (−7.3, −6.6 vs. −7.5). HAV 3Cpro is one of the main targets for the development of anti-HAV medications [32]. The discovery of natural molecules with biological activity is crucial for the identification of new drug candidates and the study of biological systems. Furthermore, limonene formed seven alkyl bonds with His44, Cys172, His145, and Met29 and showed the highest docking score of −6.9 kcal/mol with the Z10325150 compound for HAV 3C protease. All these results revealed that, notably, the hexane extract showed interactions with CDKS and HAV3C protease. These interactions may provide an important foundation for its development as a natural viral defense candidate. To build in silico results with in vivo activities, more research is required.

## 4. Materials and Methods

### 4.1. Plant Material

The whole plant of *Annona glabra* (Annonaceae) was collected from Mazhar Botanic Garden, Egypt, in January 2020. The plant was identified by Mrs. Trease Labib, a senior botanist specializing in plant taxonomy, Orman Garden, Giza, Egypt, as well as by comparison with reference herbarium specimens. At the Pharmacognosy Department, Faculty of Pharmacy, Ain Shams University, Cairo, Egypt, a voucher specimen (PHG-P-AG-381) was deposited.

### 4.2. Preparation of the Crude Extract

One kilogram of the air-dried plant was ground into fine particles and thoroughly extracted at room temperature using hexane (5 L × 3 times) for 3 days each. The resultant hexane extract was then dried completely using a rotatory evaporator at a low temperature (55 °C) and reduced pressure. This produced a sticky dark greenish extract (280 g).

### 4.3. GC-FID and GC-MS Analysis of A. glabra Hexane Extract

A Shimadzu GC with an FID detector and an HP-5 fused silica capillary column (30 m × 0.25 mm i.d., film thickness 0.25 µm) were used to perform the GC-FID analysis. The initial oven temperature was 50 °C (with a hold for 2 min and then programmed to 300 °C at 5 °C/min). Both the injector and the detector were at a temperature of 280 °C. Helium (1.0 mL/min) was employed as the carrier gas. A split ratio of 1:15 and an automatic sample injection (1 µL) was employed. Peak area normalization was used to determine the percentages that represented the relative proportions of the hexane extract’s constituents. GC-MS analysis was implemented on a Shimadzu GCMS-QP 2010 (Koyoto, Japan) equipped with an Rtx-5MS capillary column (30 m × 0.25 mm i.d. × 0.25 µm film thickness) (Restek, Bellefonte, Pennsylvania, USA). A quadrupole mass spectrometer was directly coupled to the capillary column (SSQ 7000; Thermo-Finnigan, Bremen, Germany). The injector temperature was 250 °C, and the oven temperature was kept at 50 °C for 2 min (isothermal), then programmed to 300 °C at 5 °C/min and kept constant at 300 °C for 5 min (isothermal). As a carrier gas, helium was used with a constant flow rate of 1.41 mL/min. Diluted samples (1% *v*/*v*) were injected with a split ratio of 1:15, and the injected volume was 1 µL. The MS operating parameters were as follows: ion source temperature: 200 °C, interface temperature: 280 °C, EI mode: 70 eV scan range: 35–500 amu. Components were identified based on their mass spectral data and Kovats retention indexes (KI). According to a homologous series of n-alkanes (C8–C28) injected under the same circumstances, retention indexes were calculated. The peak area percent of each compound relative to the area percent of the entire FID chromatogram (100%) was calculated.

### 4.4. Preparation of Stock Solution of the Plant Hexane Extract

We dissolved 1 g of the extract in 10 mL of Eagle’s minimal essential medium (MEM), sterilized the mixture, and stored it at stock concentrations of 100 mg/mL. For use in the in vitro investigations, further dilutions were prepared in a cell culture medium.

### 4.5. In Vitro Cytotoxicity Assay (Viability Assay)

Six human cancer cell lines were employed to test the cytotoxicity of the hexane leaf extract using the MTT assay [45].

#### 4.5.1. Cancer Cell Lines and Culture

Six human cancer cell lines were provided by the Laboratory of Virology, Microbiology Division, Faculty of Medicine (for Girls), Al-Azhar University, Cairo, Egypt. The cell lines originated from a variety of malignancies, including those of the colon (CACO-2, ATB-37), liver (HepG-2), breast (MCF-7, HTB-22), lungs (A-549), prostate (PC-3), and pancreas (PANC-1). Except for the MCF-7 cell line, which was cultured in DMEM media, other cell lines were grown in RPMI-1640 growth medium. In tissue culture flasks in an incubator set at 37 °C with 95% relative humidity and 5% CO_2_ gas atmosphere, both growth mediums (pH 7.2) were supplemented with 10% FCS, 1% penicillin (100 U/mL), and streptomycin (100 µg/mL).

#### 4.5.2. Determination of Cytotoxicity by MTT Assay

The MTT [3-(4,5-dimethylthiazole-2-yl)-2,5-diphenyl tetrazollium bromide] technique was used to assess the cytotoxicity [45]. The 96-well tissue culture plate was seeded with 1 × 10^5^ cells/mL (100 µL/mL) and incubated at 37 °C for 24 h to form a complete monolayer sheet. The growth medium was then decanted from the 96-well microtiter plates, and 0.1 mL of each dilution (made by two-fold dilutions in RPMI medium with 2% serum maintenance medium) was examined in different wells, leaving 3 wells as control, receiving only a maintenance medium. The plates were incubated at 37 °C in an atmosphere with 95% relative humidity and 5% CO_2_ gas. After 2 days, 20 μL of MTT solution (5 mg/mL in PBS, BIO BASIC INC, Markham, Canada) prepared in cell culture medium was added to each well; then, the wells were placed on a shaking table for 5 min to fully include the MTT into the media, and the plates were cultured for 4 hours to enable the metabolization of the MTT. Each well received 200 μL of DMSO to dissolve the formazan crystals after the MTT solution had been removed without disturbing the cells (MTT metabolic product). After giving the plates a light shake (150 rpm/5 min), the crystals were entirely disintegrated. At a wavelength of 560 nm, using a microplate reader, the optical density (OD) of each well was calculated.

### 4.6. Antiviral Assays

#### 4.6.1. Cell Culture and Viruses

For the antiviral experiments, Vero cells isolated from the kidney of an African green monkey (CCL-81; American Type Culture Collection, USA) were cultured in MEM containing fetal calf serum (FCS; 10% *v*/*v*), penicillin (100 U/mL), L-glutamine (2 nM), and streptomycin (100 μg/mL). Cells were multiplied for up to 4 weeks before being cultured as a cell line stock at 37 °C in a humidified environment containing 5% CO_2_. The Laboratory of Virology, Microbiology Department, Faculty of Medicine (for Girls), Al-Azhar University, Cairo, Egypt, kindly donated HAV and HSV1.

#### 4.6.2. Virus Stock Preparation

Each virus was used to multiply in Vero cells by infecting a monolayer of confluent Vero cells in 75 cm^2^ culture flasks and letting them adsorb for one hour. The infected cells were then covered with 20 mL of 2% MEM (maintenance media) and incubated until a full cytopathogenic effect (CPE) was seen daily for up to 4–6 days. Nonadherent particles were then rinsed off using 2% MEM. This process was performed twice, after which the plaque formation assay [46,47] was used to calculate the challenge dosage of each virus, and each viral harvest was kept at a temperature of −20 °C until use.

#### 4.6.3. Cytotoxicity Assay

The tested material was diluted ten times in MEM with FCS, going from 100 mg/mL to a 10-6 dilution, to determine the extract’s maximum nontoxic concentration (MNTC). Confluent Vero cells were then treated with 0.2 mL of the dilution in four of the wells of a 96-well Falcon plate (Falcon; Corning, USA). In addition to evaluating MNTC, acyclovir (Sigma-Aldrich, St. Louis, MO, USA) was dissolved in distilled water. At 37 °C, the plates were incubated. A daily inverted microscope examination of the cells was conducted to ascertain the minimum concentration necessary to cause changes in cell morphology. Even after 7 days, there were no morphological alterations between the treated and control Vero cells at the MNTC. As previously mentioned, a Colorimetric MTT assay (Bio Basic Inc., Markham, Canada) was carried out [45].

#### 4.6.4. Antiviral Protocols

The virus’s challenge dose (CDV) was introduced into cultures of Vero cells that received treatment using the extract’s MNTC and its serial dilution in accordance with three protocols in order to examine the mechanism of the antiviral activity of the plant extract [48,49,50,51]. Each protocol was independently repeated three times to ensure consistency, and the mean of the three experiments for each protocol was then given.

##### Protocol A: Virus Pretreatment (Anti-Infective Activity) [51]

The virucidal activity of the extract was examined using this procedure. The challenge virus was subjected to an equal volume of a nonlethal dilution (MNTC) of the tested extract (1:1 *v*/*v*) for one hour at 37 °C. Then, 0.5 mL of media containing the extract and an equivalent volume of the CDV viral suspension of each virus were placed on top of confluent monolayers of Vero cells in 96-well flat-bottomed microtiter plates. The viral-induced CPE was measured using the MTT colorimetric test for 72 h under an inverted microscope after the plates were incubated at 37 °C for 60 min following virus adsorption.

##### Protocol B: Postinfection Treatment (Anti-Replicative Activity) [48,50]

This technique examined how the extract affected the spread of viruses. The CDV (100 µL) of each virus was incubated with a confluent layer of Vero cells in 96-well plates. A layer of agarose containing the (MNTC) of the tested extract was applied to the cells after the cells had been adsorbed for 60 min. After 72 h, CPEs were found on the monolayer. Using the formazan absorbance value utilized in the MTT inclusion experiment, as was said earlier, the viability of both infected and healthy cells was evaluated [45]. Additionally, the IC_50_, or 50% inhibitory concentration, was computed.

##### Protocol C: Cell Pretreatment (Protective Activity) [49,50]

By preventing adhesion to the cell surface, this approach was utilized to check for viral penetration into the host cells. Confluent Vero cell monolayers were pretreated for about 48 h on a 96-well flat-bottomed microtiter plate using a medium containing the tested extract (MNTC). The cells were then challenged with the CDV of each virus after being rinsed twice with phosphate-buffer saline. The monolayer was covered with agarose solution containing the culture media and allowed to incubate after 60 min of adsorption at 37 °C. The virus-induced CPE was graded, as previously stated, after 72 h.

Three wells in each treatment were left uninoculated to serve as a negative control, while three additional wells were infected with MNTC of the antiviral drug acyclovir (positive control).

### 4.7. Molecular Docking

The Protein Data Bank provided the crystal structures of CDK2 (pdb ID.: 2a4l) [52], and HAV 3C protease (pdb ID.: 2cxv) [53] was accomplished utilizing AutoDock 4.2 [54]. The preparation of ligands and protein files, grid, and docking parameters files was conducted following the reported method [55]. The protein and the ligand were prepared and saved as pdbqt files from AutoDock software. A 3D grid box of 60 × 60 × 60 Å size with the space of 0.37 Å was placed at 100, 101, and 79 Å for docking into CDK2 and at 30.0, 0.24, and 0.27 Å for docking into HAV 3C protease. Biovia Discovery Studio 4.5. was used in the evaluation and visualization of the docking outcomes.

### 4.8. Statistical Analysis

Data of the *Annona* extract and doxorubicin (positive control) cytotoxicity against a number of cell lines including Vero, HepG-2, Caco-2, Pc-3, Panc-1, Mcf-7, and A-549 have been investigated. The antiviral activity of *Annona* extract and Acyclovir (positive control) were also investigated against HAV and HSV1 viruses. A trio of protocols (A, B, and C) was used to combat HAV, HSV1, and Newcastle viruses. All analyses were executed using Minitab 20 and SPSS 28. Before performing any statistical analyses, the data were cleaned. The data were also reviewed for missing data and typographical errors. Three replicates for each level were used to obtain descriptive statistics such as mean, standard deviation (SD), standard error (SE), minimum (min), first quartile (Q1), third quartile (Q3), median, and maximum (max). The outcomes of various groups were compared using inferential statistics. The box-cox transformation for non-normal dependent variables was implemented whenever necessary using the optimal λ approach, and all variable parametric assumptions were tested. R^2^ results showed a value of over 97%, while the adjusted R^2^ showed a value of over 95% for all cytotoxicity results, antiviral activity results showed an R^2^ value of over 65% and adjusted R^2^ over 60%, and the Newcastle virus results showed low R^2^ and adjusted R^2^. Under the fit general linear model, one-way and two-way analyses of variance were used for a number of comparisons (ANOVA). Results indicated a good fit for several models, and after data transformation, normal residual probability plots displayed a linear attitude for all studies. *p*-values were deemed significant at α < 0.05. Using the Tukey test for pairwise comparisons, a post hoc analysis of all group interactions was conducted. Results of the post hoc analyses are shown as letters, with groups sharing the same letter being nonsignificant different from one another and groups with distinct letters expressing statistically different characteristics.

## 5. Conclusions

The goal of the current inquiry was to identify different bioactive chemicals from the hexane leaf extract of *Annoa glabra* by GC-MS analysis. Tritriacontane, limonene, and 13,17-dimethyltritriacontane are the main substances in charge of the various pharmacological and therapeutic activities. We have additionally offered proof of the hexane leaf extract of *A. glabra* for its antiviral and cytotoxic activities.

This study showed that the plant extract exhibited a high variation of the cytotoxic effects against tested tumor cell lines. The reported activity was detected against the colon, lung cancer cell lines. Antiviral activity is not widely reported from *Annona* species. In this study, it was proved that an *Annona glabra* hexane extract had a pronounced protective and virucidal antiviral activity against HSV1 and mild protective antiviral activity against HAV. In addition, moderate anti-replicative activity was exerted by the plant extract against the HSV1 virus. Limonene showed the highest docking score for both HSV1 and HAV. Our research suggests that the plant could help us create trustworthy and potent medications to fight viral infections and cancer. For the development of new medication formulations, additional studies to evaluate their bioactivity and clinical trials are required.

## Figures and Tables

**Table 1:** GC–MS analysis of *Annona glabra* hexane extract.

Peak No.	Compounds	*t*_R_	Molecular Formula	KI (Cal.)	KI (Rep)	Area %	Method of Identification
**1.**	**Limonene**	**9.897**	**C_10_H_16_**	**1028**	**1029**	**18.97**	KI, MS
**2.**	*E*-Nerolidyl isobutyrate	31.515	C_19_H_32_O_2_	1833	1826	3.61	KI, MS
**3.**	Unidentified	35.768	-	2048	-	1.97	-
**4.**	Phytol	37.047	C_20_H_40_O	2115	2116	3.75	KI, MS
**5.**	*n*-Pentacosane	43.697	C_25_H_52_	2498	2500	1.54	KI, MS
**6.**	Hexacosane	45.273	C_26_H_54_	2598	2600	1.69	KI, MS
**7.**	Heptacosane	46.792	C_27_H_56_	2698	2700	2.46	KI, MS
**8.**	Octacosane	48.255	C_28_H_58_	2798	2800	2.14	KI, MS
**9.**	Nonacosane	49.667	C_29_H_60_	2897	2900	4.94	KI, MS
**10.**	Triacontane	51.034	C_30_H_62_	2998	3000	1.59	KI, MS
**11.**	Untriacontane	52.358	C_31_H_64_	3098	3100	2.63	KI, MS
**12.**	Vitamin E	53.068	C_29_H_50_O_2_	3152	3149	2.04	KI, MS
**13.**	**Tritriacontane**	**54.968**	**C_33_H_68_**	**3289**	**3300**	**30.23**	KI, MS
**14.**	**13,17-dimethyl-tritriacontane**	**55.994**	**C_35_H_72_**	**3352**	**3358**	**22.44**	KI

*t*_R_: retention time; u.i.; unidentified. Values are expressed as relative area percentages; the major components are highlighted in bold. (Values expressed as relative area percentages to the total identified components.) KI (Cal.): Calculated Kovat index; KI (Rep): Reported Kovats index.

**Table 2:** Cytotoxicity of *Annona* extract against Vero, Caco-2, HepG-2, Panc-1, Mcf-7, Pc-3, and A-549 cell lines in parameters of IC_50_ values (M ± SE) and SI.

	Vero	CACO-2		HepG-2		PANC-1		MCF-7		PC-3		A-549	
	IC_50_	IC_50_	SI	IC_50_	SI	IC_50_	SI	IC_50_	SI	IC_50_	SI	IC_50_	SI
AE	179.88 ± 0.28 A	47 ± 0.74 A	3.82	57.01 ± 0.85 A	3.10	57.34 ± 0.77 A	3.10	80.31 ± 4.13 A	2.20	81.86 ± 3.26 A	2.20	56.82 ± 0.92 A	3.10
Doxo.	34.26 ± 0.55 B	31.91 ± 0.81 B	1.07	5.4 ± 0.22 B	6.00	19.07 ± 0.2 B	1.60	15.48 ± 0.84 B	2.00	32.9 ± 1.74 B	1.04	23.39 ± 0.43 B	1.40

Doxo.: doxorubicin; AE; *Annona* extract. Groups that share letters are non-significantly different, while different letters express significant differences. SI; selectivity index = IC_50_ value normal cell/IC_50_ value cancer cell.

**Table 3:** Analyzing the cytotoxicity of the sample in Vero cells.

	ug/mL	O.D.			Mean O.D.	SD. E.	Viability %	Toxicity %	IC_50_
Vero	---	0.482	0.458	0.473	0.471	0.007	100	0	
Extract	1000	0.015	0.016	0.015	0.015333	0.000333	3.255484784	96.74451522	22.94
	500	0.018	0.016	0.017	0.017	0.000577	3.609341826	96.39065817	
	250	0.016	0.018	0.018	0.017333	0.000667	3.680113234	96.31988677	
	125	0.019	0.02	0.018	0.019	0.000577	4.033970276	95.96602972	
	62.5	0.034	0.021	0.039	0.031333	0.005364	6.652512385	93.34748762	
	31.25	0.106	0.112	0.135	0.117667	0.008838	24.98230715	75.01769285	
	15.62	0.345	0.322	0.316	0.327667	0.008838	69.56829441	30.43170559	
	7.81	0.48	0.461	0.472	0.471	0.005508	100	0	
Acyclovir	1000	0.038	0.048	0.04	0.042	0.003055	10.9947644	89.0052356	360.92
	500	0.092	0.105	0.129	0.108667	0.010837	28.44677138	71.55322862	
	250	0.274	0.271	0.246	0.263667	0.008876	69.02268761	30.97731239	
	125	0.314	0.293	0.326	0.311	0.00644	81.41361257	18.58638743	
	62.5	0.365	0.388	0.391	0.381333	0.008212	99.82547993	0.17452007	
	31.25	0.371	0.381	0.388	0.38	0.004933	99.47643979	0.523560209	

O.D.: Optical density: SD. E.: Standard deviation error.

**Table 4:** Antiviral efficacy of the *Annona glabra* extract versus HAV and HSV1 vs. acyclovir (M ± SE).

ID	Antiviral Activity %	
	HAV	HSV1
Acyclovir	46.17 ± 1.67 A	83.76 ± 5.67 A
AE	24.26 ± 9.77 B	**75.78 ± 7.51 A**

AE; *Annona* extract. Groups that share letters are non-significantly different, while different letters express significant differences.

**Table 5:** Antiviral efficacy of the *Annona glabra* extract versus HSV1 vs. acyclovir, and protocols in parameters of cell viability values (M ± SE).

ID	Antiviral Activity %		
	Protocol A	Protocol B	Protocol C
Acyclovir	83.76 ± 5.67 AB	92.69 ± 1.32 A	68.44 ± 7.62 B
AE	**70.91 ± 7.02 AB**	61.91 ± 3.51 B	**79.55 ± 1.67 AB**

AE; *Annona* extract. Groups that share letters are non-significantly different, while different letters express significant differences.

**Table 6:** Antiviral efficacy of the *Annona* extract versus HAV vs. acyclovir, and protocols in parameters of antiviral activity % values (M ± SE).

ID	Antiviral Activity %		
	Protocol A	Protocol B	Protocol C
Acyclovir	46.17 ± 1.67 A	54.8 ± 11.7 A	36.89 ± 6.61 AB
AE	36.81 ± 2.67 AB	20.13 ± 3.1 B	48.08 ± 3.46 A

AE; *Annona* extract. Groups that share letters are non-significantly different, while different letters express significant differences.

**Table 7:** Types of interactions of tritriacontane, limonene, and 13,17-dimethyltritriacontane within CKD-2 and HAV 3C protease active site.

Cyclin-Dependent Kinase 2 (Pdb; 2a4l)		
Cpd.	Amino Acids, Bond Type, Distance in (Å)	Docking Energy Scores in kcal/mol
Roscovitine	Gly13, Carbon Hydrogen Bond, 3.71 Asp145, Carbon Hydrogen Bond, 3.16 Phe80, Pi-Sigma, 3.64 Ala31, Alkyl, 3.50 Ala144, Alkyl, 3.75 Val64, Alkyl, 4.30 Val18, Alkyl, 4.84 Phe80, Pi-Alkyl, 4.37 Val18, Pi-Alkyl, 4.61 Leu134, Pi-Alkyl, 5.41 Val18, Pi-Alkyl, 4.57 Lys33, Pi-Alkyl, 4.86 Ala144, Pi-Alkyl, 4.91	−7.5
Tritriacontane	Lys88, Alkyl, 3.84 Lys89, Alkyl, 89	−6.6
Limonene	Ala144, Alkyl, 5.48 Val18, Alkyl, 5.12 Leu134, Alkyl, 4.82 Phe80, Pi-Alkyl, 4.17 Lys33, Alkyl, 4.08 Val64, Alkyl, 4.08 Ala31, Alkyl, 3.89 Ala144, Alkyl, 3.55 Phe80, Pi-Sigma, 3.49	−7.5
13,17-dimethyltritriacontane	Ala31, Alkyl, 3.72 Ile10, Alkyl, 4.67 Leu134, Alkyl, 5.17 Ile10, Alkyl, 5.04 Lys89, Alkyl, 4.02 Phe82, Pi-Alkyl, 4.36	−7.3
Hepatitis A Virus 3C Protease (Pdb; 2cxv)		
Cpd.	Amino acids, Bond type, Distance in (Å)	Docking energy scores in kcal/mol
Z10325150	His44, Hydrogen Bond, 2.16 Val28, Pi-Sigma, 3.52 Val28, Alkyl, 3.66 His145, Pi-Alkyl, 4.57 Val28, Pi-Alkyl, 5.07	−6.9
Tritriacontane	Ala193, Alkyl,3.88 His44, Pi-Alkyl, 4.34 Try143, Pi-Alkyl, 5.27	−6.3
Limonene	Cys172, Alkyl, 4.84 Met29, Alkyl, 5.15 Met29, Alkyl, 4.40 Cys172, Alkyl, 3.67 His44, Pi-Alkyl, 5.24 His44, Pi-Alkyl, 4.73 His145, Pi-Alkyl, 4.90	−6.9
13,17-dimethyltritriacontane	Val144, Alkyl, 4.90 Lys146, Alkyl, 3.64 Val28, Alkyl, 4.84	−6.7

## Data Availability

Data are available upon request from the authors.

## References

[1] Elkousy R.H., Said Z.N.A., Abd El-Baseer M.A., Abu El Wafa S.A. (2021). Antiviral activity of castor oil plant (Ricinus communis) leaf extracts. J. Ethnopharmacol..

[2] Cochrane C.B., Raveendran Nair P.K., Melnick S.J., Resek A.P., Ramachandran C. (2008). Anticancer effects of Annona glabra plant extracts in human leukemia cell lines. Anticancer Res..

[3] Mostafa N.M., Abd El-Ghffar E.A., Hegazy H.G., Eldahshan O.A. (2018). New Methoxyflavone from *Casimiroa sapota* and the Biological Activities of Its Leaves Extract against Lead Acetate Induced Hepatotoxicity in Rats. Chem. Biodivers..

[4] Ghoneim A.I., Eldahshan O.A. (2012). Anti-apoptotic effects of tamarind leaves against ethanol-induced rat liver injury. J. Pharm. Pharmacol..

[5] El-Nashar H.A.S., Mostafa N.M., Eldahshan O.A., Singab A.N.B. (2022). A new antidiabetic and anti-inflammatory biflavonoid from *Schinus polygama* (Cav.) Cabrera leaves. Nat. Prod. Res..

[6] Abdelghffar E.A., El-Nashar H.A.S., Al-Mohammadi A.G.A., Eldahshan O.A. (2021). Orange fruit (Citrus sinensis) peel extract attenuates chemotherapy-induced toxicity in male rats. Food Funct..

[7] Lúcio A.S., Almeida J.R., Da-Cunha E.V., Tavares J.F., Barbosa Filho J.M. (2015). Alkaloids of the Annonaceae: Occurrence and a compilation of their biological activities. Alkaloids Chem. Biol..

[8] Badrie N., Schauss A.G., Watson R.R., Preedy V.R. (2010). Soursop (Annona muricata L.). Bioactive Foods in Promoting Health.

[9] Kalidindi N., Thimmaiah N.V., Jagadeesh N.V., Nandeep R., Swetha S., Kalidindi B. (2015). Antifungal and antioxidant activities of organic and aqueous extracts of Annona squamosa Linn. Leaves. J. Food Drug Anal..

[10] Bhattacharya A., Chakraverty R. (2016). The pharmacological properties of Annona squamosa Linn: A Review. Int. J. Pharm. Eng. (IJPE).

[11] Doe P., Iddrisu A., Lartey P., Elijah K., Issaka S., Enock D.A. (2019). Evaluation of the anti-diarrheal activity of the ethanolic seed extract of Annona muricata. J. Phytopharmaco..

[12] Bhardwaj R., Pareek S., Sagar N.A., Vyas N., Murthy H.N., Bapat V.A. (2019). Bioactive Compounds of Annona. Bioactive Compounds in Underutilized Fruits and Nuts.

[13] Eva González-Trujano M., Tapia E., López-Meraz L., Navarrete A., Reyes-Ramírez A., Martínez A. (2006). Anticonvulsant effect of Annona diversifolia Saff. and palmitone on penicillin-induced convulsive activity. A behavioral and EEG study in rats. Epilepsia.

[14] Zorofchian S., Moghadamtousi, Rouhollahi E., Karimian H., Fadaeinasab M., Abdulla M.A., Kadir H.A. (2014). Gastroprotective activity of Annona muricata leaves against ethanol-induced gastric injury in rats via Hsp70/Bax involvement. Drug Des. Dev. Ther..

[15] Verma A., Ajay Kumar P., Kavitha D., Anurag K.B. (2011). Anti denaturation and antioxidant activities of Annona cherimola in-vitro. Int. J. Pharm. Boil. Sci..

[16] Adeyemi D.O., Komolafe O.A., Adewole O.S., Obuotor E.M., Adenowo T.K. (2008). Antihyperglycemic activities of Annona muricata (Linn). Afr. J. Tradit. Complement. Altern. Med..

[17] Bhalke R.D., Chavan M.J. (2011). Analgesic and CNS depressant activities of extracts of Annona reticulate Linn. Bark. J. Phytopharm..

[18] Betancur-Galvis L., Saez J., Granados H., Salazar A., Ossa J. (1999). Antitumor and antiviral activity of Colombian medicinal plant extracts. Mem. Inst. Oswaldo Cruz.

[19] Grzybowski A., Tiboni M., Silva M.A., Chitolina R.F., Passos M., Fontana J.D. (2013). Synergistic larvicidal effect and morphological alterations induced by ethanolic extracts of Annona muricata and Piper nigrum against the dengue fever vector Aedes aegypti. Pest Manag. Sci..

[20] Zhang Y.H., Peng H.Y., Xia G.H., Wang M.Y., Han Y. (2004). Anticancer effect of two diterpenoid compounds isolated from Annona glabra Linn. Acta Pharmacol. Sin..

[21] Singh T.P., Singh R.K., Malik P. (2014). Analgesic and Anti-inflammtory Activities of Annona squamosa Linn bark. J. Sci. Innov. Res..

[22] Eldahshan O.A. (2013). Rhoifolin: A potent antiproliferative effect on cancer cell lines. Br. J. Pharm. Res..

[23] Hien N.T., Nhiem N.X., Yen D.T., Hang D.T., Tai B.H., Quang T.H., Tuan Anh H.L., Kiem P.V., Minh C.V., Kim E.J. (2015). Chemical constituents of the Annona glabra fruit and their cytotoxic activity. Pharm. Biol..

[24] Liu X.X., Alali F.Q., Pilarinou E., McLaughlin J.L. (1998). Glacins A and B: Two novel bioactive mono-tetrahydrofuran macetogenins from Annona glabra. J. Nat. Prod..

[25] Chang F.R., Yang P.Y., Lin J.Y., Lee K.H., Wu Y.C. (1998). Bioactive Kaurane Diterpenoids from Annona glabra. J. Nat. Prod..

[26] Tatar O., Sriamornsak P., Dass C.R. (2013). Doxorubicin: An update on anticancer molecular action, toxicity, and novel drug delivery systems. Pharm. Pharmacol..

[27] Gamal El-Din M.I., Youssef F.S., Ashour M.L., Eldahshan O.A., Singab A.N.B. (2018). Comparative Analysis of Volatile Constituents of Pachira Aquatica Aubl. and Pachira glabra Pasq., their Anti-Mycobacterial and Anti-Helicobacter pylori Activities and their Metabolic Discrimination using Chemometrics. J. Essent. Oil Bear. Plants.

[28] Younis I.Y., El-Hawary S.S., Eldahshan O.A., Abdel-Aziz M.M., Ali Z.Y. (2021). Green synthesis of magnesium nanoparticles mediated from *Rosa floribunda* charisma extract and its antioxidant, antiaging, and antibiofilm activities. Sci. Rep..

[29] Schonbrunn E., Betzi S., Alam R., Martin M.P., Becker A., Han H., Francis R., Chakrasali R., Jakkaraj S., Kazi A. (2013). Development of Highly Potent and Selective Diaminothiazole Inhibitors of Cyclin-Dependent Kinases. J. Med. Chem..

[30] Peyressatre M., Prevel C., Pellerano M., Morris M.C. (2015). Targeting Cyclin-Dependent Kinases in Human Cancers: From Small Molecules to Peptide Inhibitors. Cancers.

[31] Chohan T.A., Qian H.Y., Pan Y.L., Chen J.Z. (2016). Molecular Simulation Studies on the Binding Selectivity of 2-Anilino-4-(Thiazol-5-yl)-Pyrimidines in Complexes with CDK2 and CDK7. Mol. Biosyst..

[32] Zhou J., Wang D., Xi Y., Zhu X., Yang Y., Lv M., Luo C., Chen J., Ye X., Fang L. (2017). Assessing activity of Hepatitis A virus 3C protease using a cyclized luciferase-based biosensor. Biochem. Biophys. Res. Commun..

[33] Ben-Shabat S., Yarmolinsky L., Porat D., Dahan A. (2020). Antiviral effect of phytochemicals from medicinal plants: Applications and drug delivery strategies. Drug Deliv. Transl. Res..

[34] Chen C.H., Hsieh T.J., Liu T.Z., Chern C.L., Hsieh P.Y., Chen C.Y. (2004). Annoglabayin, a novel dimeric kaurane diterpenoid, and apoptosis in hep G2 cells of annomontacin from the fruits of Annona glabra. J. Nat. Prod..

[35] Liu X.X., Alali F.Q., Pilarinou E., McLaughlin J.L. (1999). Two bioactive mono-tetrahydrofuran acetogenins, annoglacins A and B, from Annona glabra. Phytochemistry.

[36] Anh H.T., Hien N.T., Hang D.T., Ha T.M., Nhiem N.X., Hien T.T., Thu V.K., Thao D.T., Van Minh C., Kiem P.V. (2014). Ent-Kaurane diterpenes from Annona glabra and their cytotoxic activities. Nat. Prod. Commun..

[37] O’Brien J.J., Campoli-Richards D.M. (1989). Acyclovir. An updated review of its antiviral activity, pharmacokinetic properties, and therapeutic efficacy. Drugs.

[38] Jingjing Y., Lei Z., Ren L., Yunzheng Y., Jiye Y., Qingsong D., Xiaojia G., Wei L., Yuexiang L., Miaomiao L. (2022). In vitro and in vivo antiviral activity of Maqian (Zanthoxylum myriacanthum var. pubescent) essential oil and its major constituents against strains of influenza virus. Ind. Crops Prod..

[39] Fadilah N.Q., Jittmittraphap A., Leaungwutiwong P., Pripdeevech P., Dhanushka D., Mahidol C., Ruchirawat S., Kittakoop P. (2022). Virucidal Activity of Essential Oils From Citrus x aurantium L. Against Influenza A Virus H1N1: Limonene as a Potential Household Disinfectant Against Virus. Nat. Prod. Commun..

[40] Astani A., Schnitzler P. (2014). Antiviral activity of monoterpenes beta-pinene and limonene against herpes simplex virus in vitro. Iran. J. Microbiol..

[41] Konappa N., Udayashankar A.C., Krishnamurthy S., Pradeep C.K., Chowdappa S., Jogaiah S. (2020). GC–MS analysis of phytoconstituents from Amomum nilgiricum and molecular docking interactions of bioactive serverogenin acetate with target proteins. Sci. Rep..

[42] Viegas D.J., Edwards T.G., Bloom D.C., Abreu P.A. (2019). Virtual screening identified compounds that bind to cyclin dependent kinase 2 and prevent herpes simplex virus type 1 replication and reactivation in neurons. Antivir. Res..

[43] Schang L.M., Bantly A., Schaffer P.A. (2002). Explant-induced reactivation of herpes simplex virus occurs in neurons expressing nuclear cdk2 and cdk4. J. Virol..

[44] Schang L.M., St Vincent M.R., Lacasse J.J. (2006). Five years of progress on cyclin-dependent kinases and other cellular proteins as potential targets for antiviral drugs. Antivir. Chem. Chemother..

[45] Takeuchi H., Baba M., Shigeta S. (1991). An application of tetrazolium (MTT) colorimetric assay for the screening of anti-herpes simplex virus compounds. J. Virol. Methods.

[46] Reed L., Muench H. (1938). A simple method of estimating 50% endpoints. Am. J. Hyg..

[47] Dulbecco R., Vogt M. (1954). Plaque formation and isolation of pure lines with poliomyelitis viruses. J. Exp. Med..

[48] Kaul T., Middleton E., Ogra P. (1985). Antiviral effect of flavonoids on human viruses. J. Med. Virol..

[49] Kaye S. (2000). Antiviral methods and protocols. J. Antimicrob. Chemother..

[50] Gescher K., Kühn J., Hafezi W., Louis A., Derksen A., Deters A., Lorentzen E., Hensel A. (2011). Inhibition of viral adsorption and penetration by an aqueous extract from *Rhododendron ferrugineum* L. as antiviral principle against herpes simplex virus type-1. Fitoterapia.

[51] Flechas M., Ocazionez R., Stashenko E. (2017). Evaluation of in vitro antiviral activity of essential oil compounds against dengue virus. Pharmacogn. Rev. J..

[52] De Azevedo W.F., Leclerc S., Meijer L., Havlicek L., Strnad M., Kim S.H. (1997). Inhibition of cyclin-dependent kinases by purine analogues: Crystal structure of human cdk2 complexed with roscovitine. Eur. J. Biochem..

[53] Yin J., Bergmann E.M., Cherney M.M., Lall M.S., Jain R.P., Vederas J.C., James M.N. (2005). Dual modes of modification of hepatitis A virus 3C protease by a serine-derived beta-lactone: Selective crystallization and formation of a functional catalytic triad in the active site. J. Mol. Biol..

[54] Morris G.M., Huey R., Lindstrom W., Sanner M.F., Belew R.K., Goodsell D.S., Olson A.J. (2009). AutoDock4 and AutoDockTools4: Automated docking with selective receptor flexibility. J. Comput. Chem..

[55] Trott O., Olson A.J. (2010). AutoDock Vina: Improving the speed and accuracy of docking with a new scoring function, efficient optimization, and multithreading. J. Comput. Chem..

